# Defect Profile Estimation from Magnetic Flux Leakage Signal via Efficient Managing Particle Swarm Optimization

**DOI:** 10.3390/s140610361

**Published:** 2014-06-12

**Authors:** Wenhua Han, Jun Xu, Ping Wang, Guiyun Tian

**Affiliations:** 1 College of Automation Engineering, Shanghai University of Electric Power, Shanghai 200090, China; E-Mail: daniel_xiaoxu@163.com; 2 College of Automation Engineering, Nanjing University of Aeronautics and Astronautics, Nanjing 210016, China; E-Mail: zeit@263.net; 3 School of Electrical and Electronic Engineering, Newcastle University, Newcastle upon Tyne, NE1 7RU, UK; E-Mail: g.y.tian@ncl.ac.uk

**Keywords:** magnetic flux leakage, profile estimation, efficient managing particle swarm optimization, high dimension optimization problem

## Abstract

In this paper, efficient managing particle swarm optimization (EMPSO) for high dimension problem is proposed to estimate defect profile from magnetic flux leakage (MFL) signal. In the proposed EMPSO, in order to strengthen exchange of information among particles, particle pair model was built. For more efficient searching when facing different landscapes of problems, velocity updating scheme including three velocity updating models was also proposed. In addition, for more chances to search optimum solution out, automatic particle selection for re-initialization was implemented. The optimization results of six benchmark functions show EMPSO performs well when optimizing 100-D problems. The defect simulation results demonstrate that the inversing technique based on EMPSO outperforms the one based on self-learning particle swarm optimizer (SLPSO), and the estimated profiles are still close to the desired profiles with the presence of low noise in MFL signal. The results estimated from real MFL signal by EMPSO-based inversing technique also indicate that the algorithm is capable of providing an accurate solution of the defect profile with real signal. Both the simulation results and experiment results show the computing time of the EMPSO-based inversing technique is reduced by 20%–30% than that of the SLPSO-based inversing technique.

## 1. Introduction

**Figure 1.**
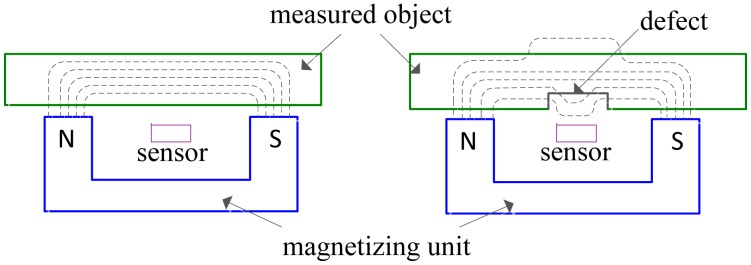
Magnetic flux leakage testing method for object without defect (**left**) and object with defect (**right**).

As one of the nondestructive testing techniques [1–3], magnetic flux leakage (MFL) is widely applied to the inspection of defects in oil and gas pipelines and aboveground storage tanks made of ferromagnetic material. In the MFL testing, MFL signal are acquired by an array of hall-effect sensors closely disposed above the surface of measured object when the object is magnetically saturated by strong permanent magnets shown in Figure 1. By processing MFL signal with a certain signal processing method, the corresponding defect profile or parameters of the defect shape can be estimated [4].

**Figure 2.**
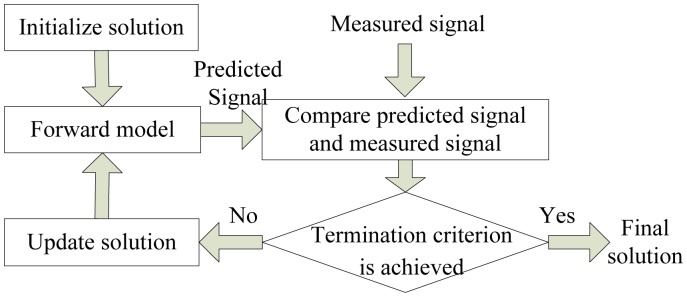
The framework of inversing techniques.

Due to the possible situation that different defects generate similar magnetic field distributions and the uneven distribution of the defect's leakage magnetic field, if the magnetic field is given, there may be several defect profiles. So the results are always morbid; that is to say, they are lack of uniqueness and continuity, provided that directly using MFL signal to estimate defect profile. For these reasons, inversing techniques became the most commonly used methods and have been of utmost interest in the community where the chosen solution minimizes the sum of the squared differences between the measured MFL signal and the signal predicted by the forward model [5]. The framework of inversing techniques for MFL inspection is presented in Figure 2.

For a well-performed inversing technique to estimate defect profile, suitable forward model and iterative procedure are indispensable. A physical model is usually employed as the forward model which mainly involve three classes: heuristic models (e.g., artificial neural network [6,7]), analytical models (e.g., dipole model [8,9]), and numerical models (e.g., finite element method [10,11]). Compared with numerical models and analytical models, the heuristic models are faster but less accurate. In heuristic models, radial-basis function neural network (RBFNN) has been successfully used to profile estimation.

An inversing procedure is often regarded as solving an optimization problem, and many optimization algorithms have been applied to the inversing techniques, such as gradient descent algorithm [6,7] and genetic algorithm (GA) [12,13]. It's proved that the efficiency of the iterative approach applied to solve optimization problem determines the computing time and solution accuracy of inversing technique.

Particle swarm optimization (PSO), firstly proposed by Eberhart and Kennedy in 1995 [14], is one of the most important swarm intelligence algorithm. As PSO uses a relatively simple mechanism that mimics swarm behaviors such as fish in a school, birds in a flock to adaptively guide the particles to search for globally optimal solution, it has been actively studied and applied for many academic and real world problems with promising results [15–17].

Similar to other optimized algorithm based on swarm intelligence such as GA, the standard PSO algorithm begins with a random initialization of each particle in the solution space, and then each particle iteratively searches the solution space according to only a single learning pattern by using the new velocity and the previous location which can save time and computing resources. However, due to the particles' searching strategy, the standard PSO may be lack of intelligence to cope with different complex situations. Many experiments have shown that the standard PSO may easily get trapped in a local optimum when solving complex multimodal problems [18]. In this paper, for enhancing the performance of PSO, a new variant of PSO, called efficient managing PSO (EMPSO) is proposed and used to the inversing technique as an iterative approach.

This paper is organized as follows: Section 2 describes the background of the EMPSO-based inversing technique. Section 3 presents a description of EMPSO and its flowchart, and Section 4 shows the results of six benchmark functions optimized by EMPSO. Section 5 describes the new inversing technique based on EMPSO. Finally, Section 6 reports both simulation results and experimental results by estimating defect profiles with the new inversing technique and Section 7 presents the conclusions.

## 2. Background of EMPSO-Based Inversing Technique

In early work [19], parameters of defect shape are estimated by using the inversing technique based on damping-boundary-based PSO (DBPSO). The effectiveness of the DBPSO was verified by the application to defect parameters estimation, the results of which demonstrated that DBPSO-based inversing technique is promising for solving MFL signal inverse problems.

With the requirement of visualization, estimating profile of defect directly draws more and more attention, instead of the shape parameters estimation. Different from estimation of defect parameters, in order to estimate defect profile accurately, the defect profile is necessary to be separated into many uniform discrete values, and this produces the dimension of the problem. The estimation of defect profile is formulated to a high dimension optimization problem. So finding a variant of PSO with higher optimizing capability is urgent.

In order to improve PSO's searching ability and efficiency, many PSO variants have been proposed. Among these variants, efficient population utilization strategy for PSO (EPUS-PSO) is incorporated with population manager to eliminate redundant particles and hire new ones or maintain particle numbers and two built-in sharing strategies to enhance sharing among all particles [20]. Cooperative coevolving particle swarm optimization (CCPSO2) adopts a new PSO location update rule that relies on Cauchy and Gaussian distributions to sample new points in the search space and a scheme to dynamically determine the coevolving subcomponent sizes of the variables [21]. In self-learning particle swarm optimizer (SLPSO), each particle has a set of four strategies to cope with different situations in the solution space [22]. Although these algorithms have been widely applied to various optimization problems, finding a kind of PSO algorithm with the capability to optimize high complex problems with efficient search behavior, rapid convergence and outstanding optimization results is still an active area of research [23]. It has been proved that by applying CCPSO2 and EPUS-PSO to inversing technique, the estimated profiles are not successfully close to the true profiles. Inspired by the above several PSO variants, EMPSO is proposed in this paper.

## 3. EMPSO

For efficiently handling high dimension optimization problems like defect profile estimation, EMPSO is presented, which includes three modifications to make the process more efficient from three different aspects. In order to strengthen exchange of information among particles, particle pair model (PPM) was built and introduced to PSO. For more efficient searching when facing different landscapes of problems, velocity updating scheme including three velocity updating model was also proposed. In addition, for achieving more rational utilization of computing resources and more chances to search optimum solution out, automatic particle selection for re-initialization was implemented.

### 3.1. PPM

In standard PSO algorithm and its variants, each particle is considered as a relatively independent agent, which has indirect information exchange via *gbest* (global best location) when velocity is updated or by applying some methods like searching-range-sharing strategy in EPUS-PSO [20]. However, it's difficult for such information exchange methods to effectively find potential better solutions. For example, solution-sharing strategy in EPUS-PSO denotes a unique sharing rate for each particle to decide if randomly selecting two another particles' *pbest* (personal best location) from the particle swarm and then choosing the better one to update velocity, this strategy really accomplishes information exchange, but in fact, after applying solution-sharing strategy, the location of a particle may be even worse than its previous one, because the selected *pbest* of another particle could make the particle further from optimum solution.

In order to avoid above situation, a new model for information exchange, called PPM is introduced by imitating some animals which form a team by several units to forage. By applying PPM, particle swarm will be divided into several particle pairs containing two particles, the two particles in the same particle pair have the common *pbest*, which means *pbest* of a particle pair will be updated as long as either of the particle pair finishes location updating, but the two particles have their own freedom to finish velocity and location updating. Thus, particles of particle pair complement each other's advantages and will be more likely to gain better solution than two irrelevant particles in traditional ways.

### 3.2. Velocity Updating Scheme

In standard PSO, velocity update of each particle is affected by its own *pbest* and *gbest* at the same time. In fact, each particle is not simply influenced by *gbest*; all the neighbors' *lbest* (local best location) are used to modify the velocity of a particle [18]. So far, most variants of PSO used two of the above three models to update particles' velocity, but it's generally believed that the pulls of the three models is different.

In details, *pbest*, *gbest* and *lbest* are inclined to exploitation, convergence and exploration respectively, so if each particle simultaneously learns from two models, the algorithm may suffer from the disadvantages of the two models. For example, for standard PSO, after several iterations, particles will gather in several clusters, or even just one cluster, which is probably the local optimal solution. Each particle in the cluster may perform a local search to make evolution continue but not be able to jump out from local optimal solution to explore other better solutions. This kind of terrible situation is caused by excessive convergence, so an *lbest* model should be added to guide particles to search new spaces. If a *pbest* model and an *lbest* model are both used, it's likely for particles to have inadequate search in their own nearby regions of *pbest* position.

For these reasons, *gbest*, *pbest* and *lbest* models are used independently to update velocity. A *gbest* model is a suitable velocity updating model for convergence, especially at the final phase of iterations, with enough search of solution space, and it's urgent for all particles to converge on optimum search results. For a certain particle, its *pbest* is the best solution so far, a better solution is likely near *pbest*, especially when the particle is in a slope. So for most problems, exploiting the region near *pbest* is an efficient method. For the *lbest* model, many works has been done about how to utilize information from neighbors. For example, all neighbors of a particle are used to update velocity by some certain topology, a particle searches through its neighbors in order to identify the one with the best result so far, and uses information from that one source to bias its search in a promising direction [23]. The utilization of neighbors promotes diversity of velocity updating and keeps a balance between a particle's own *pbest* and its neighbors' *pbest*.

Three velocity updating equations corresponding to the three models are shown as follows.

(1) Each particle updates velocity with its previous velocity and *gbest*:
  (1) $$v_{k}^{d}(t+1)=\omega v_{k}^{d}(t)+cr(\text{gbes}t^{d}(t)-x_{k}^{d}(t))$$
(2) Each particle updates velocity with its previous velocity and *pbest*:
  (2) $$v_{k}^{d}(t+1)=\omega v_{k}^{d}(t)+cr(\text{pbes}t_{k}^{d}(t)-x_{k}^{d}(t))$$
(3) Each particle updates velocity with its previous velocity and *lbest*:
  (3) $$v_{k}^{d}(t+1)=\omega v_{k}^{d}(t)+cr(\text{lbes}t_{k}^{d}(t)-x_{k}^{d}(t))$$

where $v_{k}^{d}(t+1)$ is the velocity in the *d*th dimension of the *k*th particle at the (*t*+1)th iteration, $x_{k}^{d}(t)$ is the location in the *d*th dimension of the *k*th particle at the (*t*)th iteration, $lbest_{k}^{d}(t)$ is the *pbest* of the better particle from two random particles except the particle itself, *r* is a randomly distributed number, *c* is a coefficient which is usually set to be 1.49, *ω* is the inertia weight.

It should be noted that at the early phase of iterations, the importance of exploration and exploitation is high and the importance of convergence is increasing along with iterations. So it's useful to classify these three learning models into two types, that is, type 1 with (1) for converging and type 2 with (2–3) for exploring and exploiting. The probability of using type 2 to update velocity begins with the maximum and decreases along with iterations. The probability of choosing type 1 is exactly opposite. In every generation, decide whether to select type 1 or type 2 by comparing selection ratio *p*_1_ with a random number uniformly distributed from 0 to 1. If type 2 is selected, in the same way, selection ratio *p*_2_, also compared with a random number uniformly distributed from 0 to 1, is used to decide whether to select a particle's own *pbest* or *lbest*. *p*_1_ is updated along with iterations and *p*_2_ is a constant:

(4) $$p_{1}(t)=p_{\max}-(p_{\max}-p_{\min})\frac{t}{t_{\max}}$$

where *p*_max_ and *p*_min_ are the maximum and minimum of *p*_1_ which are usually set to be 0.85 and 0.3. *p*_2_ is a probability constant set to be 0.8.

### 3.3. Particle Re-Initialization Strategy

For PSO, if a particle cannot find a better solution to update its *pbest* in several consecutive iterations, it may accomplish adequate search around *pbest* and be trapped into the local minimum [18]; If the *gbest* has not been updated in several consecutive iterations, it also may accomplish adequate search around *gbest* and there is no better solution than *gbest* around all particles' *pbest*. It is time-consuming and inefficient in the two situations, and avoiding them as far as possible can enhance searching ability. Based on it, particles in the above two situations can reinitialize their locations and velocities like particle initialization at the beginning of iterations, thus particles are able to search new parts of the solution space.

However, it is difficult to effectively implement this idea. This is because it is scarcely possible to exactly know when particles are in the two situations. So the two activating thresholds (*T*_1_, *T*_2_) , which both are positive integer numbers, denote maximum permissible times for particle not finding any better solution to update *pbest* and for particle swarm not updating *gbest*, respectively. In details, if a particle's *pbest* has not been updated over *T*_1_ iterations, the location and velocity will be reinitialized, randomly assigned with values in the solution space and speed range, respectively. If the *gbest* of the particle swarm has not been updated over *T*_2_ iterations, both two particles of the particle pair with worst *pbest* in the particle swarm will also be reinitialized with random locations and velocities. Revived particles will never be affected by their past parameters and search for better solution in the new locations. It is worthwhile to note that *T*_1_, *T*_2_ should be assigned with suitable numbers, too large thresholds cannot enhance the search ability effectively, too small thresholds will mistakenly reinitialize many particles which are not in the above two situations.

**Figure 3.**
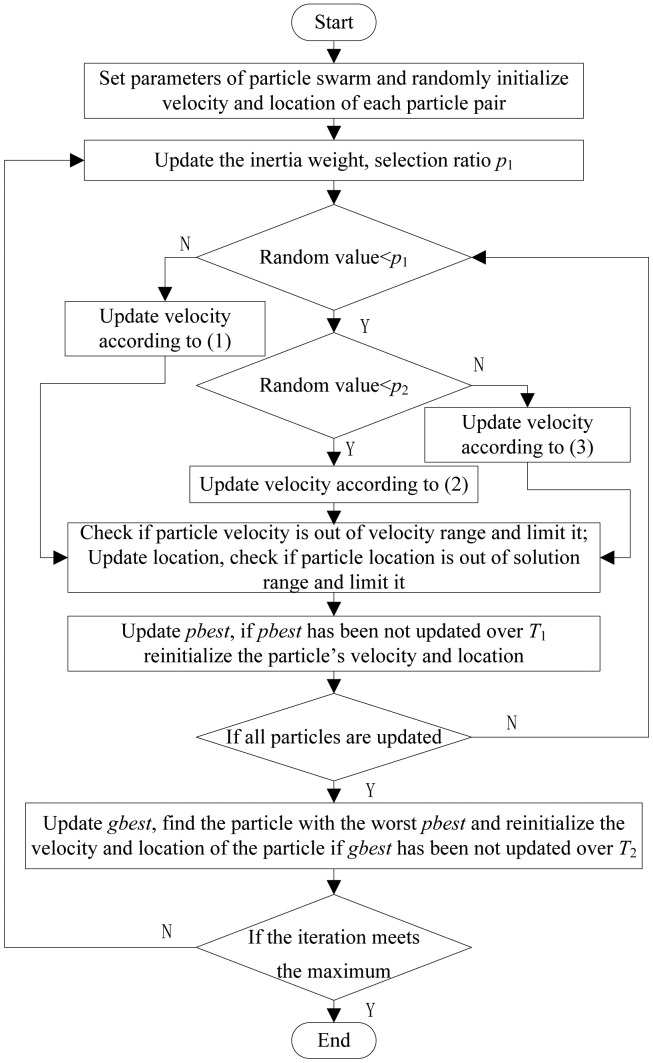
The flowchart of EMPSO.

Above all, PPM, velocity updating scheme and particle re-initialization strategy are proposed to improve the performance of PSO for high dimensional global optimization problem. The complete flowchart of the EMPSO is shown in Figure 3. After dividing particle swarm to several particle pairs and initializing all the parameters, each particle adaptively selects one of the three velocity updating models to update velocity and location. Next, check whether the particle needs re-initialization. Repeat these steps until the iteration meets maximum.

## 4. Optimization Results of Six Benchmark Functions

To investigate how EMPSO performs in different types of problems and compare it with SLPSO and EPUS-PSO, several benchmark functions including traditional functions and shifted function are chosen [18].

### 4.1. Benchmark Functions

All benchmark functions used in this paper are shown as follows:

- Griewank function:
  (5) $$f_{1}(\vec{x})=1/4000{\sum_{i=1}^{n}\left(x_{i}-100\right)}^{2}-\prod_{i=1}^{n}\cos(\frac{x_{i}-100}{\sqrt{i}})+1$$
- Rastrigin function:
  (6) $$f_{2}(\vec{x})=\sum_{i=1}^{n}\left(x_{i}^{2}-10\cos2\pi x_{i}+10\right)$$
- Rosenbrock function:
  (7) $$f_{3}(\vec{x})=\sum_{i=1}^{n}\left(100(x_{i+1}^{2}-x_{i})+{\left(x_{i}-1\right)}^{2}\right)$$
- Schwefel function:
  (8) $$f_{4}(\vec{x})=418.9829n+\sum_{i=1}^{n}-x\sin(\sqrt{\left|x_{i}\right|})$$
- Schwefel_2_21 function:
  (9) $$f_{5}(\vec{x})=\max_{i=1}\left|x_{i}\right|$$
- Shifted Schwefel_1_2 function:
  (10) $$f_{6}(\vec{x})=\sum_{i=1}^{n}{\left(\sum_{j=1}^{i}z_{j}\right)}^{2},\vec{z}=\vec{x}-\vec{o}$$

$\vec{o}=[\begin{array}{cccc} o_{1} & o_{2} & \ldots & o_{n} \end{array}]$: the shifted global optimum.

Different functions own different search range, these functions' search range and global optimum are listed in Table 1.

The fitness value of the six benchmark functions means the function value of the best particle in PSO algorithm. The fitness value changes with algorithm iteration. The final optimization result of an algorithm is the fitness value of the last iteration.

### 4.2. Parameter Settings and Initialization of the Three Algorithms

The experiments compared three variants of PSO, including EMPSO, SLPSO and EPUS-PSO on six benchmark functions with 100 dimensions (100-D). All the PSO variants were implemented with MATLAB R2011b. The inertia weight and acceleration coefficients of each peer algorithm are presented in Table 2, which is exactly the same as that used in the original paper. The pairs of the swarm size and maximum iterations for solving 100 dimension problems are set to (40, 300,000). For EMPSO, T1, T2 are set with 20 and 25.

### 4.3. Optimization Results

**Figure 4.**
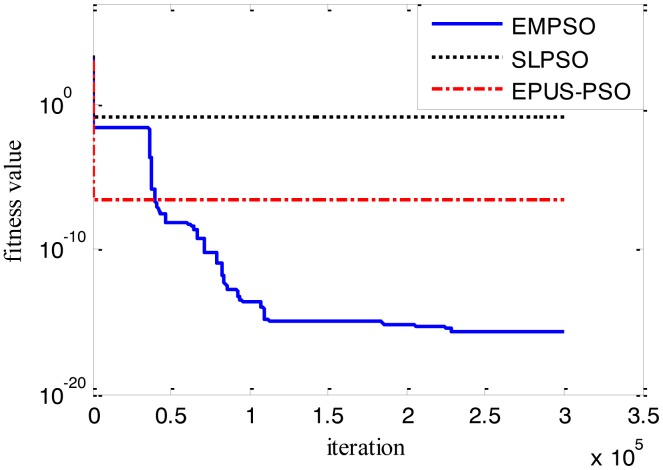
100-D Griewank function (*f_1_*).

**Figure 5.**
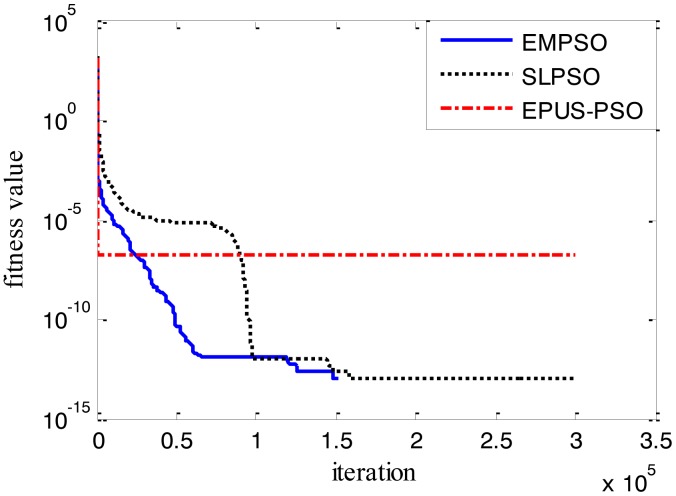
100-D Rastrigin function (*f_2_*).

**Figure 6.**
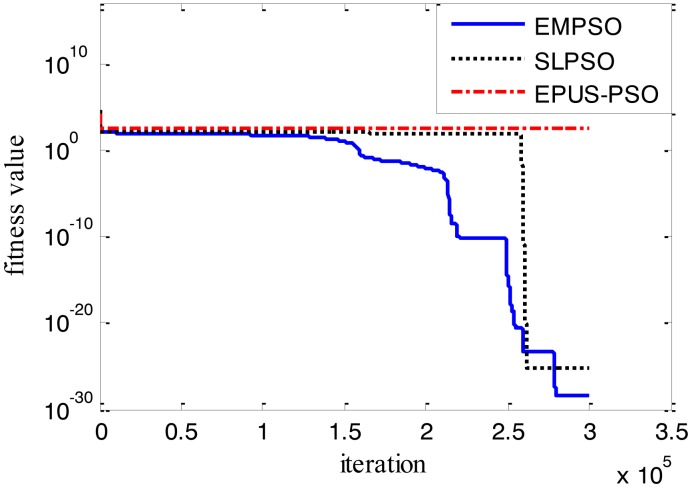
100-D Rosenbrock function (*f_3_*).

**Figure 7.**
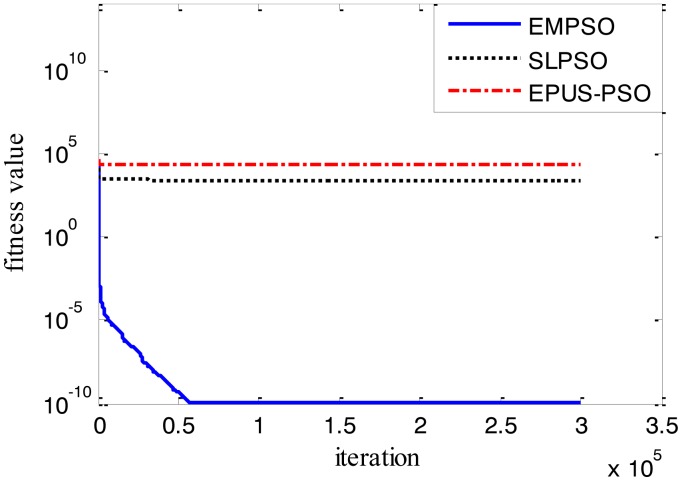
100-D Schwefel function (*f_4_*).

**Figure 8.**
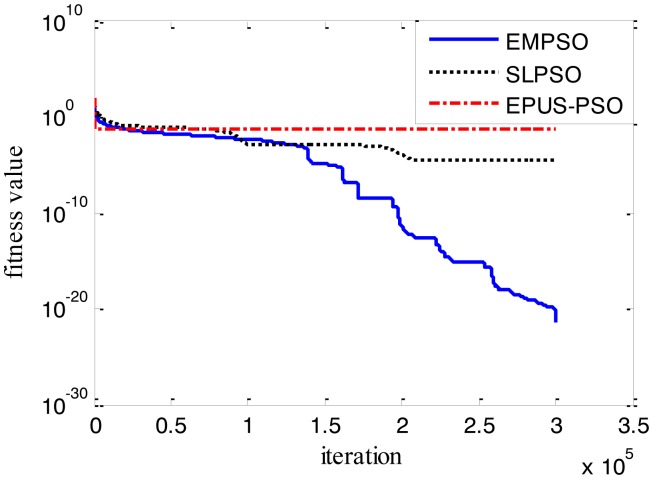
100-D Schwefel_2_21 function (*f_5_*).

**Figure 9.**
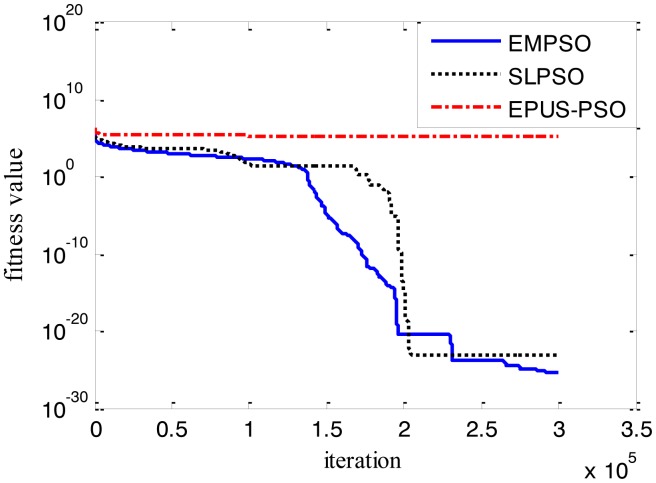
100-D shifted Schwefel_1_2 function (*f_6_*).

Results of the 100-D test functions: the best solutions of the three algorithms on the six benchmark functions with 100 dimensions are shown on Table 3, where the best result on each problem among all algorithms is shown in bold. With the increasing dimensions of test functions, the optimization will be more complicated. Even though the number of particles and maximum of generations are increased, the results may not be as good as them in the 30-D test functions, but still quite good. The performance of EMPSO is still better than SLPSO and EPUS-PSO, which is partly attributable to the more significant effect of the particle pair model and three different velocity updating methods. Figures 4, 5, 6, 7, 8 and 9 show the fitness change curves of the optimum solution for these algorithms with six test functions. It can be seen that the convergence of EMPSO is still faster than that of SLPSO and EPUS-PSO.

The optimization results show EMPSO is capable to optimize different types of 100-D problems. In this paper, the inversing technique for defect profile estimation is a kind of 100-D problem, so EMPSO is introduced to the inversing technique as iterative algorithm.

## 5. Inversing Technique Based on EMPSO

In the new inversing technique based on EMPSO, a radial basis function neural network (RBFNN) is selected as the forward model to predict MFL signals from a defect profile which has been proved to be feasible to accurately model the forward process [6]. The iterative procedure is achieved by EMPSO to minimize a cost function that represents the difference between the predicted signals of the neural networks forward model and the measured signals. The cost function is shown:

(11) $$F=\sum_{j=1}^{M}{\left(p_{j}-y_{j}\right)}^{2}$$

where *M* is the dimension of the MFL signals, $P=[\begin{array}{cccc} p_{1} & p_{2} & \ldots & p_{M} \end{array}]$ is the measured MFL signals, $Y=[\begin{array}{cccc} y_{1} & y_{2} & \ldots & y_{M} \end{array}]$ is the MFL signals predicted by RBFNN.

**Figure 10.**
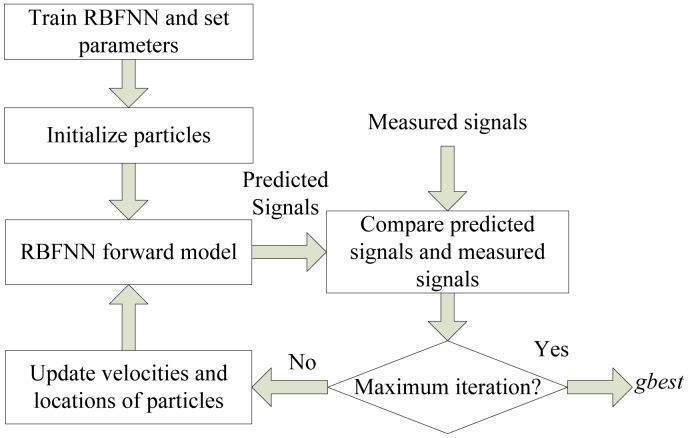
The framework of the new inversing technique.

For the new inversing technique, the first step is training RBFNN and initializing the parameters and particles' locations and velocities. Then the values of cost function are calculated after the new locations are transduced into the predicted MFL signals by forward model. All particles' locations (also defect profiles) and velocities are updated. The cost of predicted signals becomes smaller and smaller along with the iterations of EMPSO. Finally, when the termination criterion is achieved, the predicted MFL signal of the optimum profile will be close to the measured signal. The framework of inversing technique based on EMPSO is shown in Figure 10.

## 6. Simulation Results and Experimental Results

**Figure 11.**
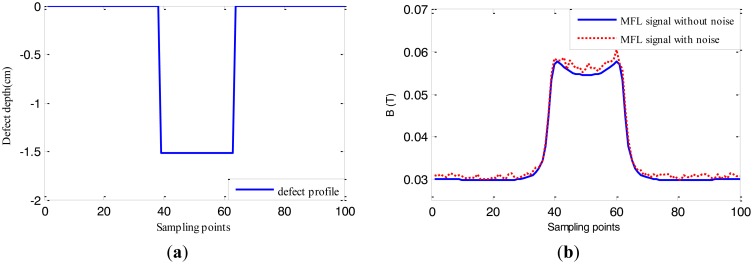
The profile and MFL signal for the defect (12.7 cm width, 1.524 cm deep). (**a**) The defect profile; (**b**) The MFL signal.

In this paper, firstly MFL data [6] simulated by software are used to verify the performance of the inversing technique based on EMPSO including 240 2-D defect samples with varying widths and depths. 210 defect samples are used to train RBFNN and the remaining 30 are for profile estimation. An example of defect samples is shown in Figure 11, where the true profile of the defect (12.7 cm width, 1.524 cm deep) is denoted by solid line in Figure 11a and its MFL signal in the presence of 5% noise and the one without noise are denoted by dotted line and solid line, respectively, in Figure 11b. The size of the sampling point interval is 0.508 cm. RBFNN with 100 input nodes and 100 output nodes are used as forward model, spread of radial basis functions is 1 × 10^−12^.

Self-learning PSO (SLPSO) [22] is also applied, which has a superior performance in comparison with several other peer algorithms. For comparing the inversing technique based on EMPSO with the one based on SLPSO, MFL signal without noise and with 5% noise are used to estimate defect profiles. *T_1_*, *T_2_* are set to be 20 and 25. Both EMPSO and SLPSO have a population size set at 80 particles. The inertia weight was decreased linearly from 0.9 to 0.4 over 20,000 iterations and 50,000 iterations for estimation of MFL signal without noise and with 5% noise, respectively. The solution range of 100 dimensions is from −2.159 to 0 cm.

**Figure 12.**
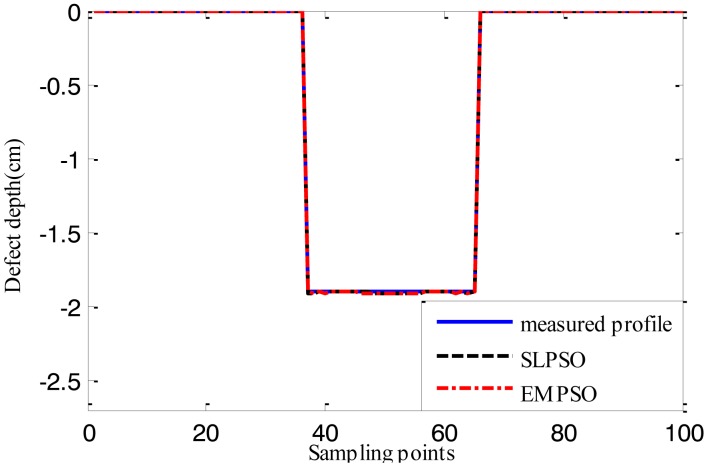
The profiles estimated by the two inversing techniques (MFL signal without noise, 14.732 cm width, 1.905 cm depth).

**Figure 13.**
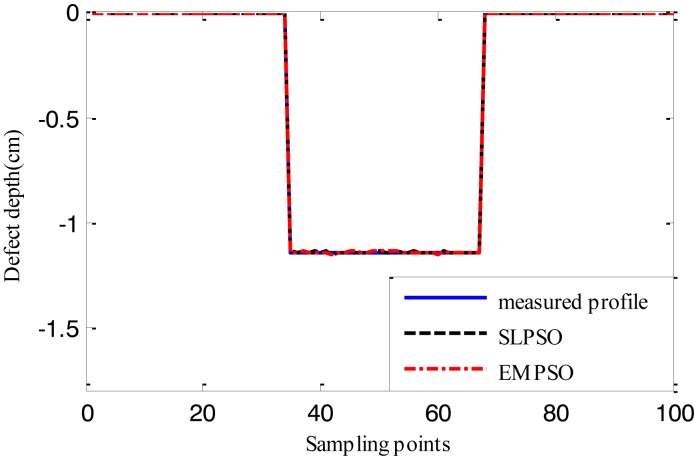
The profiles estimated by the two inversing techniques (MFL signal without noise, 16.764 cm width, 1.143 cm depth).

**Figure 14.**
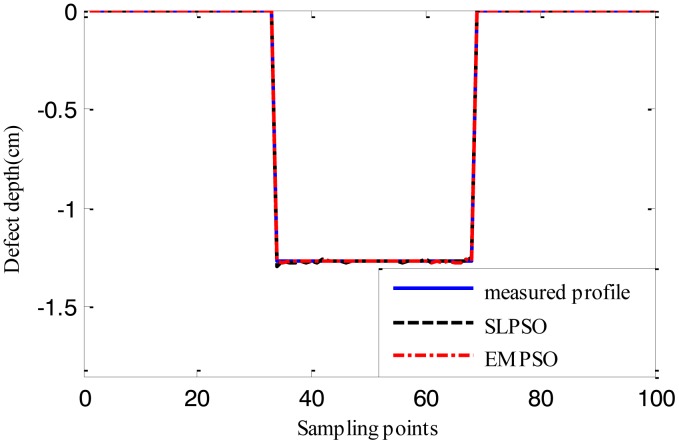
The profiles estimated by the two inversing techniques (MFL signal without noise, 17.78 cm width, 1.27 cm depth).

Figures 12, 13 and 14 show the estimated defect profiles by processing MFL signal without noise. In details, the solid line, dotted line and chain line denote the measured profile, profile estimated by the inversing technique based on SLPSO and profile estimated by the one based on EMPSO, respectively. The values of cost function of the two estimated profiles and their computing time are shown in Table 4.

Both estimated profiles are close to the measured profile, but the differences between them are not obvious, and it is proved from the values of cost function for the estimated results that the inversing technique based on EMPSO outperforms the one based on SLPSO and is less time-consuming.

**Figure 15.**
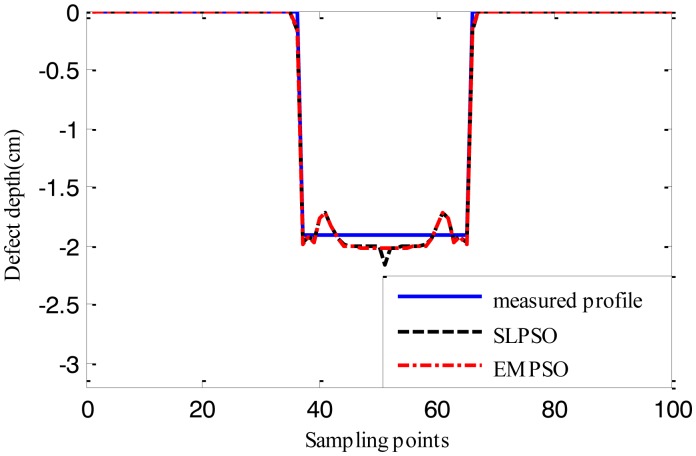
The profiles estimated by the two inversing techniques (MFL signal with 5% noise, 14.732 cm width, 1.905 cm depth).

**Figure 16.**
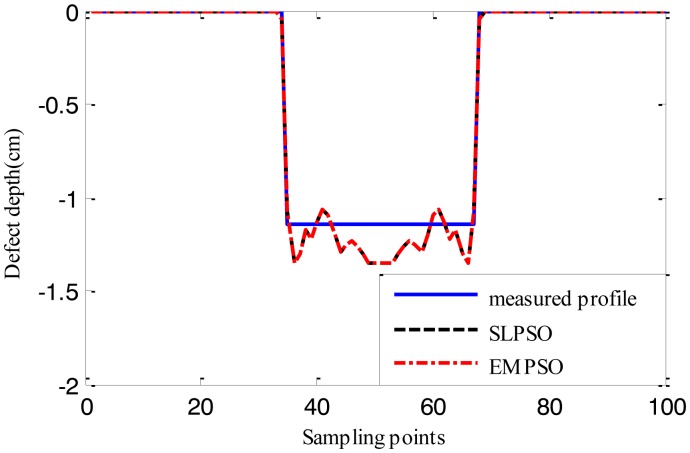
The profiles estimated by the two inversing techniques (MFL signal with 5% noise, 16.764 cm width, 1.143 cm depth).

**Figure 17.**
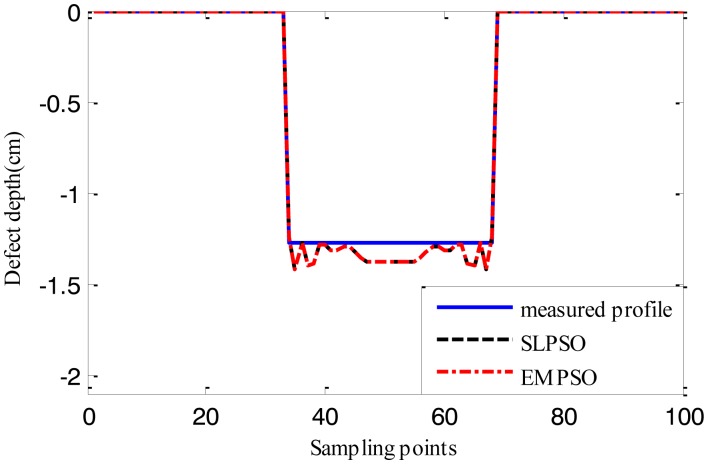
The profiles estimated by the two inversing techniques (MFL signal with 5% noise, 17.78 cm width, 1.27 cm depth).

Figures 15, 16 and 17 show the reconstructed defect profiles by processing MFL signal with 5% noise. The values of cost function of the two profiles estimated by SLPSO-based inversing technique and EMPSO-based inversing technique and their computing time are in Table 5.

Due to the existence of 5% noise in MFL signal, the estimated profiles are further from the true profile than the ones estimated from MFL signal without noise. But the results are also near the true profiles, and for EMPSO-based inversing technique, the values of cost function are better than the ones calculated by SLPSO-based inversing technique. What's more, the proposed technique also consumes less time.

**Figure 18.**
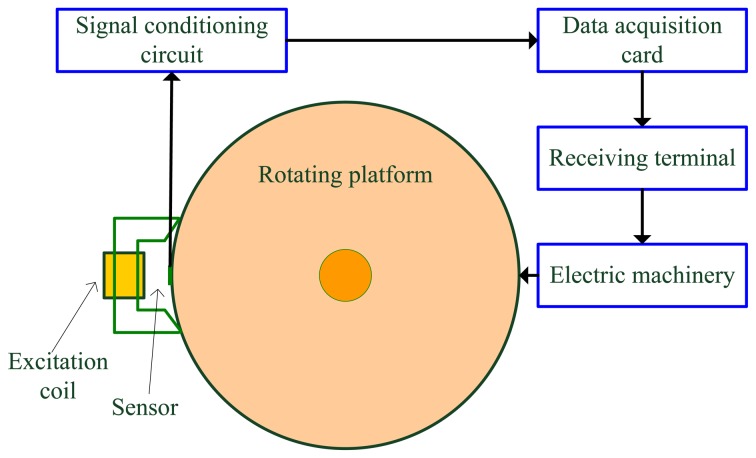
Schematic of the experimental equipment.

In order to further verify the performance of the proposed EMPSO-based inversing technique, the measured MFL data are used. The schematic of experimental equipment is shown in Figure 18.

The experimental equipment mainly includes a rotating platform, excitation coil, sensors, signal conditioning circuit, data acquisition card, receiving terminal (personal computer here) and electric machinery. Many defects are distributed on the edge surface of the rotating platform. A magnetizing yoke with an excitation coil is used to generate a magnetic field, the magnetic pole of which is 1 mm distance from the rotating platform. The Hall sensor probe is located at the center of the two magnetic poles of the magnetizing yoke at 0.5 mm distance from the edge surface, aiming to acquire the MFL signal. After regulated by the signal conditioning circuit, MFL signals are transmitted to the data acquisition card. Finally, the computer receives the MFL signals. In addition, the speed of the rotating platform is controlled by electric machinery.

The type of material of the top surface of the rotating platform is U71Mn. Defects with different sizes are distributed on the top surface of the rotating platform, the practical speed of which ranges from 2 to 50 m/s. The types of Hall-effect sensors and data acquisition card are UGN3503 and ADLINK DAQ 2204. As the amplitude of the MFL detecting signal is of a millivolt level and the range of data acquisition card is volt level, an AD620 instrumentation amplifier is applied to design an amplifying circuit whose amplification factor is 100. In addition, to avoid the detection device from magnetizing the rotating platform repeatedly, we lay out the magnetization reversal device opposite to the detection device.

**Figure 19.**
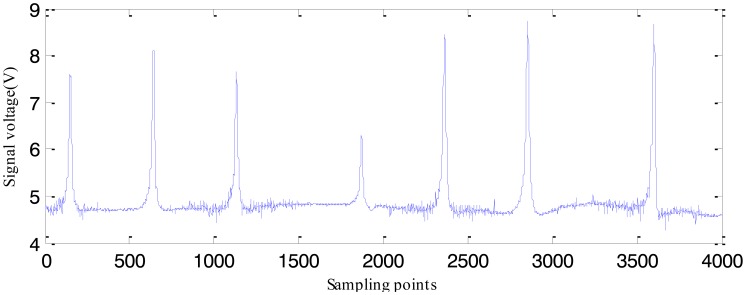
The measured real MFL signal.

Figure 19 shows the experimental MFL signals gathered by sensors on groove defects. As we can see, different from simulated MFL signals, the experiment MFL signals includes noise signals which mainly appear when the Hall sensors acquire the signals. Before using the above signal simulated by software to estimate real profiles, the measured real MFL signal is periodically sampled and normalized to staying the same level with the simulated signal. Here the two inversing techniques based on EMPSO and SLPSO are applied to real defect profile estimation.

**Figure 20.**
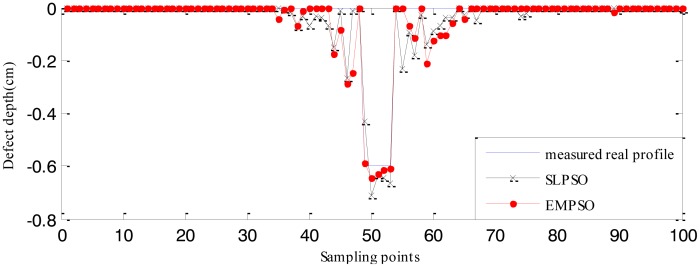
Comparison between the real profile and estimated profiles (defect 1).

**Figure 21.**
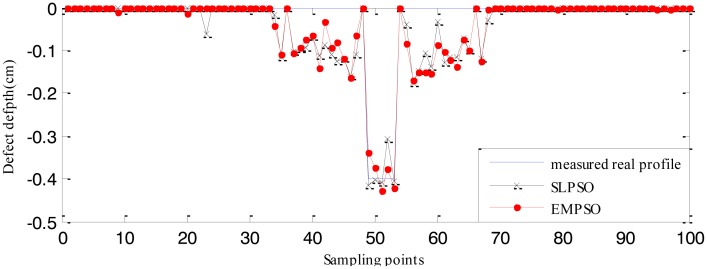
Comparison between the real profile and estimated profiles (defect 2).

The RBFNN trained by 210 defect samples is regarded as the forward model. The estimated defect profiles for defect 1 (0.04 cm width, 0.6 cm depth) and defect 2 (0.04 cm width, 0.4 cm depth) from the measured real signals are shown in Figures 20 and 21. The computation time and cost function values are listed in Table 6. From Tables 4 , 5 and 6, it can be seen that the computation time of the EMPSO-based inversing technique is reduced by 20%–30% compared to that of the SLPSO-based inversing technique, and the cost value of the former is less than or the same to that of the latter, so the overall performance of the proposed EMPSO-based inversing technique is better than the SLPSO-based inversing technique.

The profiles estimated by the EMPSO-based inversing technique are still closer to the real profiles than those of the SLPSO-based inversing technique, and the EMPSO-based inversing technique consumes less time. Overall, the profiles estimated from measured real signal aren't as good as the ones estimated from simulated signals which have more errors. The main reason for this could be the measurement environment for real MFL signal is more complex. The experiments show the EMPSO-based inversing technique is suitable for estimating profiles from real signals.

## 7. Conclusions

The objective of this paper was to propose a new variant of PSO called EMPSO and apply an inversing technique based on EMPSO to profile estimation of 2-D defects. Firstly, the optimization results of the selected six benchmark functions prove the good performance of EMPSO when handling 100-D problems. Then the experimental results demonstrate that the inversing technique based on EMPSO is capable of estimating 2-D defect profiles and outperforms the inversing technique based on SLPSO. With the existence of low noise in MFL signals, the estimated profiles are still close to the measured profiles. The results estimated from real MFL signals by the EMPSO-based inversing technique also indicate that the algorithm is capable of providing an accurate solution of the defect profiles with real signals. The future work will be developed from two aspects, that is applying the inversing technique to the estimation of 3-D defect profiles and largely decreasing the computing time of the inversing technique.

## Figures and Tables

**Table 1.** Search range and global optimum.

**Function Number**	**Search Range**	**Global Optimum**
*f*_1_	[–600,600]	0
*f*_2_	[–5.12,5.12]	0
*f*_3_	[–2.048,2.048]	0
*f*_4_	[–500,500]	0
*f*_5_	[–100,100]	0
*f*_6_	[–100,100]	0

**Table 2.** Parameters' settings of three variants of PSO.

**Algorithm**	**Inertia Weight**	**Acceleration Coefficients**
EMPSO	$\omega =0.9-\frac{0.5gen}{\max\_gen}$	*c*=1.49
SLPSO	$\omega =0.9-\frac{0.5gen}{\max\_gen}$	*c*=1.96
EPUS-PSO	$\omega =\frac{1}{2\ln2}$	*c*_1_=*c*_2_=0.5+ln2

**Table 3.** Final results of 100-D test functions.

**Function Number**	**EMPSO**	**SLPSO**	**EPUS-PSO**

*f*_1_	**2.22 × 10^−16^**	0.13	2.76 × 10**^−^**^7^
*f*_2_	**0**	1.14 × 10**^−^**^13^	1.73 × 10**^−^**^7^
*f*_3_	**3.86 × 10^−29^**	7.12 × 10**^−^**^26^	305.7
*f*_4_	**1.09 × 10^−10^**	2.02 × 10^3^	2.34 × 10^4^
*f*_5_	**3.91 × 10^−22^**	2.90 × 10**^−^**^5^	0.06
*f*_6_	**4.43 × 10^−26^**	6.56 × 10**^−^**^24^	1.83 × 10^5^

**Table 4.** Values of Cost Function and Computing Time.

**Inversing Methods**	**Profile Shape (cm)**	**Cost Values**	**Computing Time (s)**
SLPSO	14.732 width, 1.905 depth	3.5724 × 10^−10^	3.745 × 10^4^
	16.764 width, 1.143 depth	4.4422 × 10^−11^	4.676 × 10^4^
	17.78 width, 1.27 depth	2.3556 × 10^−11^	4.747 × 10^4^
EMPSO	14.732 width, 1.905 depth	**3.5620 × 10^−10^**	**2.207 × 10^4^**
	16.764 width, 1.143 depth	**4.1402 × 10^−11^**	**3.351 × 10^4^**
	17.78 width, 1.27 depth	**1.9449 × 10^−11^**	**3.214 × 10^4^**

**Table 5.** Values of Cost Function and Computing Time.

**Inversing Methods**	**Profile Shape (cm)**	**Cost Values**	**Computing Time (s)**
SLPSO	14.732 width, 1.905 depth	7.8196 × 10^−5^	1.712 × 10^4^
	16.764 width, 1.143 depth	6.1103 × 10^−5^	2.063 × 10^4^
	17.78 width, 1.27 depth	5.9973 × 10^−5^	1.575 × 10^4^
EMPSO	14.732 width, 1.905 depth	**7.8167 × 10^−5^**	**1.260 × 10^4^**
	16.764 width, 1.143 depth	6.1103 × 10^−5^	**1.516 × 10^4^**
	17.78 width, 1.27 depth	5.9973 × 10^−5^	**1.256 × 10^4^**

**Table 6.** Values of cost function and computing time.

**Inversing Methods**	**Experimental Samples**	**Cost Values**	**Computing Time (s)**
SLPSO	Defect 1	6.3581 × 10^−5^	1.132 × 10^4^
	Defect 2	4.7932 × 10^−5^	1.192 × 10^4^

EMPSO	Defect 1	**5.9781 × 10^−5^**	**8.740 × 10^3^**
	Defect 2	**4.3911 × 10^−5^**	**9.149 × 10^3^**
